# Fragmented QRS for Risk Stratification in Patients Undergoing First Diagnostic Coronary Angiography

**DOI:** 10.5935/abc.20160139

**Published:** 2016-10

**Authors:** Mehmet Eyuboglu, Mehmet Akif Ekinci, Suleyman Karakoyun, Ugur kucuk, Omer Senarslan, Bahri Akdeniz

**Affiliations:** 1 Avrupa Medicine Center - Turkey; 2 Alfakalp Medicine Center - Turkey; 3 Kars Kafkas University Medical School - Turkey; 4 Gulhane Military Medical Academy, Haydarpasa Training Hospital - Turkey; 5 School of Medicine, University of SIFA - Turkey; 6 Dokuz Eylul University Hospital - Turkey

**Keywords:** Coronary Artery Disease, Coronary Angiography, Electrocardiography, Fragmented QRS, SYNTAX score

## Abstract

**Background:**

Only a small proportion of patients referred for coronary angiography with suspected coronary artery disease (CAD) have the diagnosis of obstructive CAD confirmed by the exam. For this reason, further strategies for risk stratification are necessary.

**Objective:**

To investigate the relationship of the presence of fragmented QRS (fQRS) on admission electrocardiogram with angiographically detected CAD and CAD severity in patients without known vascular diseases and myocardial fibrosis, undergoing first diagnostic coronary angiography.

**Methods:**

We enrolled 336 consecutive patients undergoing coronary angiography for suspected CAD. The patients were divided into two groups according to the presence or absence of fQRS on admission. We compared the groups regarding the presence and severity of CAD.

**Results:**

Seventy-nine (23.5%) patients had fQRS on admission. There was not a statistically significant difference between patients with fQRS (41.8%) and non-fQRS (30.4%), regarding the presence of CAD (p = 0.059). However, there was a statistically significant difference between patients with fQRS and non-fQRS regarding the presence of stenotic CAD (40.5% vs. 10.5%, p<0.001) and multi vessel disease (25,3% vs. 5.1%, p<0.001). The frequency of fQRS was significantly higher in patients with SYNTAX score >22 compared to patients with SYNTAX score ≤22.

**Conclusions:**

Our findings suggest that fQRS may be an indicator of early-stage myocardial damage preceding the appearance of fibrosis and scar, and may be used for risk stratification in patients undergoing first diagnostic coronary angiography

## Introduction

Fragmented QRS complex (fQRS) is an easy-to evaluate electrocardiographic finding. It is defined as a QRS with a duration <120 ms, with notched R or S waves, without accompanying typical bundle branch block or additional wave such as RSR' pattern in two contiguous leads in one of the major coronary artery territories in the original QRS complex.^1^ The presence of fQRS on electrocardiography (ECG) is a sign of delay in ventricular conduction, associated with myocardial scarring, ischemia, and fibrosis.^2^ fQRS is an independent predictor of impaired myocardial perfusion, left ventricular dilatation and decreased ejection fraction in patients with ischemic heart disease, and is strongly correlated with adverse outcomes, arrhythmia and mortality in patients with coronary artery disease (CAD).^3-5^

Coronary angiography is the best modality to detect the presence and severity of CAD and define the coronary anatomy in patients with suspicious CAD.^6^ However, as it is an invasive procedure and not free from complications, it should be reasonably performed.^7^ Only a small proportion of patients referred for coronary angiography with suspected CAD have the diagnosis of obstructive CAD confirmed by coronary angiography,^8,9^ suggesting that better risk stratification strategies are necessary. Significance of fQRS in patients without known vascular diseases and apparent myocardial fibrosis is unknown. The aim of the present study was to investigate the relationship between fQRS complex on admission ECG and angiographically detected CAD, stenotic CAD, and CAD severity in patients without known vascular diseases undergoing first diagnostic coronary angiography.

## Methods

### Study population

A total of 439 consecutive patients with a suspicion of CAD who underwent first diagnostic coronary angiography were enrolled. All patients had a suspicious or positive treadmill test or myocardial perfusion scintigraphy. Patients with diabetes mellitus (n = 44), family history of CAD (n = 11), coronary slow flow (n=6), chronic inflammatory diseases or elevated C-reactive protein levels (n = 11), chronic kidney disease (n = 9), evidence of left ventricle hypertrophy (n = 6), known vascular disease (n=4), moderate to severe valvular heart disease (n = 4), complete or incomplete bundle-branch block and QRS duration ≥ 120 ms (n = 8) were excluded. As a result, 336 patients were included into the study. Demographic characteristics, cardiovascular risk factors and laboratory parameters were recorded on admission. Diabetes mellitus was defined as fasting plasma glucose ≥ 126 mg/dL or blood glucose ≥ 200 mg/dL at any time or treatment with antidiabetic medications. Hypertension was defined as blood pressure >140/90 mmHg or treatment with antihypertensive drugs. The patients were divided into two groups according to the presence or absence of fQRS on admission.

The study was approved by the local ethics committee and study protocol complied with the Declaration of Helsinki.

### Electrocardiography

**Figure 1:**
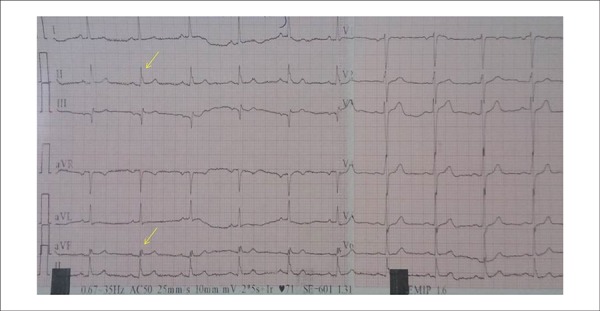
Electrocardiogram of a patient with fragmented QRS

Twelve-lead surface ECG was obtained from all patients. All ECGs were analyzed blindly by two independent cardiologists. In case of disagreement, the final decision on the presence of fQRS was achieved by mutual agreement. The fQRS was defined as a QRS with various RSR' patterns or notched R or S waves, with a duration of < 120 ms, in the absence of bundle branch block in two contiguous leads corresponding to a major coronary artery territory^1^ (Figure 1).

### Coronary angiography and SYNTAX score (SXscore)

Coronary angiography was performed by femoral or radial approach using the standard Judkins technique. Digital angiographic images were evaluated by two independent interventional cardiologists. Diameter stenosis ≥ 50% in the left main coronary artery and ≥ 70% in the other epicardial coronary arteries was accepted as critical stenosis. CAD was defined as presence of stenotic or non-stenotic atherosclerotic lesions in any coronary arteries. The number of vessels with critical luminal stenosis in coronary arteries was categorized as one or multi vessel disease (MVD). Additionally, SYNTAX score (SXscore) was used to define the extent, complexity and severity of CAD. SXscore has been developed based on angiographic characteristics, and specific functional and anatomical parameters of the atherosclerotic lesions^10-12^. The SXscore was calculated for all coronary lesions causing > 50% diameter stenosis in a vessel > 1.5 mm, based on the SXscore calculator (www.syntaxscore.com). An SXscore > 22 was defined as intermediate-high and SXScore ≤ 22 was defined as a low SXscore. All angiographic variables were calculated by two experienced interventional cardiologists who were totally blinded to the study. If there was any controversy, the final decision was made by consensus.

### Statistical analysis

All data were analyzed using SPSS 15.0 version (SPSS Inc., Chicago, Illinois). Numerical variables were expressed as mean ± SD, whereas categorical variables were expressed as percentage values. Comparisons between groups were made by using the analysis of variance, Kruskal-Wallis or Chi-square tests, as appropriate. Continuous variables were compared between the groups with Student's t-test or Mann-Whitney U test. A 2-sided p value < 0.05 was considered significant in all analyses.

## Results

The mean age of patients was 50.9 ± 3.5 years and 61.9% of patients were male. The baseline clinical and laboratory parameters of patients are shown in Table 1. On electrocardiographic evaluation, 79 (23.5%) patients had fQRS. There was no significant difference between fQRS and non-fQRS patient groups regarding age, gender, hypertension and smoking. As a result of coronary angiography, 111 patients (33%) had CAD - 34 patients had left anterior descending artery (LAD) lesions, 24 left circumflex artery (LCX) lesions and 20 right coronary artery (RCA) lesions - 59 patients (17.6%) had stenotic CAD and 33 (9.8%) patients had MVD. There was not a statistically significant difference between patients with fQRS (41,8%) and without fQRS (30,4%), regarding presence of CAD (p = 0.059). However, there was a statistically significant difference between patients with and without fQRS regarding the presence of stenotic CAD and MVD (40.5% vs. 10.5%, p < 0.001 and 25.3% vs. 5.1%, p < 0.001, respectively) (Table 2).

**Table 1:** Baseline clinical and laboratory parameters of study population

Variables	
Age, years	50.9 ± 3.5
Gender, Male,%	61.9
Hypertension,%	26.5
Smoking,%	30.1
Fragmented QRS,%	23.5
**CAD**	
LAD, %	10.1
LCX,%	7.1
RCA,%	6
MVD,%	9.8
Stenotic CAD,%	17.6
Syntax Score, median	18 ± 7.1
LDL,mg/dL	117 ± 28
TG,mg/dL	155 ± 46
HDL,mg/dL	39 ± 8
Glucose	89 ± 11
Creatinin	0.9 ± 0.18
WBC	6.7 ± 2.2
Hemoglobin	13 ± 2.3
LVEF	61 ± 4.9

CAD: coronary artery disease; LAD: left anterior descending artery; LCX: left circumflex artery; RCA: right coronary artery; MVD: multivessel disease; LDL: low-density lipoprotein; TG: triglyceride; HDL: high-density lipoprotein; WBC: white blood cell counts; LVEF: left ventricular ejection fraction.

**Table 2:** Baseline clinical and laboratory parameters of patients with and without fragmented QRS

Variables	Fragmented QRS (n = 79)	Non-fragmented QRS (n = 257)	p value
Age,years	51.4 ± 4.6	50.8 ± 3.1	0.408
Gender, Male,%	59.5	62.6	0.614
Hypertension,%	30.4	25.3	0.970
Smoking,%	38	27.6	0.079
CAD,%	41.8	30.4	0.059
MVD,%	25.3	5.1	< 0.001
Stenotic CAD,%	40.5	10.5	< 0.001
LDL,mg/dl	125 ± 34	118 ± 26	0.089
TG,mg/dl	161 ± 43	156 ± 46	0.124
HDL,mg/dl	39.1 ± 8.2	39.2 ± 8	0.979
Glucose	91 ± 14	89 ± 11	0.195
Creatinin	0.95 ± 0.25	0.9 ± 0.18	0.127
WBC	6.9 ± 2.3	6.7 ± 2.2	0.474
Hemoglobin	13 ± 2.3	13.1 ± 2.4	0.635
LVEF	59.3 ± 6	60.7 ± 5.1	0.070

CAD: coronary artery diseas; MVD: multivessel disease; LDL: low-density lipoprotein; TG: triglyceride; HDL: high-density lipoprotein; WBC: white blood cell counts; LVEF: left ventricular ejection fraction.

In the subgroup analysis, we divided the patients into two groups according to SXscore. The median SXscore value of the study group was 18±7.1; 43 patients (72.9%) were in group 1 (SXscore ≤ 22) and 16 patients (27.1%) were in group 2 (SXscore > 22). Hypertension was more frequent in group 2 (66.7%) than in group 1 (26.8%) (p = 0.004), and there was a significant difference between groups regarding LDL (113 ± 29 vs. 131 ± 32, p = 0.039). The incidence of fQRS was significantly higher in group 2 (94.4%) than in group 1 (36.6%) (p < 0.001) (Table 3). There was a statistically significant difference in the frequency of patients with and without MVD between group 1 (41.5% and 58.5%) and group 2 (88.9% and 11.1%) p=0.001, respectively.

**Table 3:** Baseline clinical and laboratory parameters of patients according to Syntax Score

Variables	Syntax Score ≤ 22 (n = 43)	Syntax Score > 22 (n = 16)	p value
Age,years	55 ± 3.9	56 ± 3.2	0.813
Gender, Male,%	82.9	88.9	0.558
Hypertension,%	26.8	66.7	0.004
Smoking,%	36.6	44.4	0.569
Syntax Score,median	14 ± 5.8	24 ± 1.5	< 0.001
Fragmented QRS,%	36.6	94.4	< 0.001
MVD,%	41.5	88.9	0.001
LDL,mg/dl	113 ± 29	131 ± 32	0.039
TG,mg/dl	158 ± 42	171 ± 50	0.309
HDL,mg/dl	39 ± 7.5	37 ± 7	0.356
Glucose	91 ± 13	98 ± 14	0.076
Creatinin	0.9 ± 0.25	0.96 ± 0.28	0.370
WBC	6.3 ± 1.7	6.4 ± 1.6	0.818
Hemoglobin	13.4±2.2	13.1±2.8	0.710
LVEF	59.7±5.8	57.6±6.2	0.230

MVD: multivessel disease, LDL: low-density lipoprotein, TG: triglyceride, HDL: high-density lipoprotein, WBC: white blood cell counts, LVEF: left ventricular ejection fraction.

## Discussion

The main finding of our study was that the presence of fQRS on admission ECG was associated with higher frequency of CAD, stenotic CAD, MVD and higher SXscore in patients undergoing first diagnostic coronary angiography. fQRS is a sign of myocardial scar and it is a predictor of adverse outcomes in patients with acute coronary syndromes, CAD, structural heart diseases and arrhythmogenic syndromes.^13-16^ However, the predictive value of fQRS in terms of risk stratification is not well described in patients who are without evidence of myocardial scarring and undergo first coronary angiography due to a suspicion of CAD. We found that the incidence of fQRS was 23.5% in our study population. This finding is similar to a recently published study which investigated the incidence and prognostic value of fQRS in 10,904 middle-aged subjects with and without known cardiac diseases.^17^ The investigators found an incidence of 19.7% of fQRS, which was not associated with increased mortality in individuals without known cardiac diseases, and hence, the importance of fQRS in these patients remains a challenge. Our study included middle age subjects with normal left ventricular ejection fraction, and all factors that could be associated with myocardial fibrosis, including vascular disease, as well as systemic or inflammatory diseases were excluded. Besides, we did not include patients with diabetes, and the incidence of cardiovascular risk factors was low in the study group. Also, 42.2% of them did not have positive stress test.

Coronary angiography is widely used and is the gold standard for detecting CAD. Despite advances in the techniques used to perform coronary angiography, complications associated with invasive procedures are still a challenge.^6,7^ Furthermore, most of the patients undergoing coronary angiography have normal angiograms or non-obstructive CAD.^8,9^ Hence, further risk stratification strategies are necessary, in particular, in patients undergoing first diagnostic coronary angiography. In our study, the frequency of CAD was higher in patients with fQRS on surface ECG, and fQRS was significantly associated with higher incidence of stenotic CAD and severe CAD. These findings suggest that fQRS may be used in risk stratification in patients undergoing first diagnostic coronary angiography. fQRS is a sign of myocardial scar and ventricular conduction delay in various conditions besides CAD. It is also a sign of electrical dyssynchrony in patients with non-ischemic dilated cardiomyopathy and a narrow QRS interval.^18^ Additionally, in the absence of CAD, left ventricular hypertrophy and increased left ventricle mass are associated with higher frequency of fQRS in patients with normal left ventricular ejection fraction.^19-21^ Therefore, in our study, in addition to vascular diseases, we excluded patients with lower ejection fraction, moderate to severe valvular diseases and evidence of left ventricular hypertrophy to identify the exact predictive value of fQRS in terms of presence of CAD and CAD severity.

SXscore is a scoring system for angiographic anatomy that quantifies the complexity and severity of CAD, and indicates adverse outcomes in patients with CAD.^10,22^ It has been demonstrated that fQRS on admission ECG was associated with higher SXscore in patients with acute coronary syndrome.^23^ However, this finding had not been confirmed in patients undergoing coronary angiography for the diagnosis of CAD. Hence, we found that the frequency of fQRS was significantly higher in patients with intermediate to high SXscore (SXscore > 22) compared with patients with low SXscore (SXscore ≤ 22) in our study population.

To our knowledge, this is the first study to include such a low risk study population to investigate the relationship between fQRS and angiographic findings. Despite excluding various factors that may be associated with apparent fibrosis or scar, we found that fQRS is a predictor of the presence of CAD, and obstructive and severe CAD. Therefore, fQRS may be an indicator of early-stage myocardial damage before the appearance of fibrosis and scar.

### Study limitations

The present study has some limitations. First, the study population was relatively small that could reduce statistical power. Second, we did not perform subgroup analyses based on the localization and number of leads with fQRS. However, the main goal of the present study was to investigate the association between fQRS and the presence of CAD and CAD severity, and we were able to demonstrate a significant association between fQRS and obstructive and severe CAD in our study population.

## Conclusion

The presence of a narrow fQRS on admission ECG is significantly associated with stenotic CAD and higher SXscore in patients undergoing first diagnostic coronary angiography. Also, fQRS seems to be an indicator of obstructive or non-obstructive CAD in these patients. fQRS is a simple, easy detectable ECG parameter, and our findings suggest that it can be used for risk stratification in patients without evidence of vascular diseases or myocardial fibrosis and scar undergoing diagnostic coronary angiography.
